# Impact of an AI software on the diagnostic performance and reading time for the detection of cerebral aneurysms on time of flight MR-angiography

**DOI:** 10.1007/s00234-024-03351-w

**Published:** 2024-04-15

**Authors:** Nils C. Lehnen, Arndt-Hendrik Schievelkamp, Christian Gronemann, Robert Haase, Inga Krause, Max Gansen, Tobias Fleckenstein, Franziska Dorn, Alexander Radbruch, Daniel Paech

**Affiliations:** 1 Department of Neuroradiology, University Hospital Bonn, Rheinische Friedrich-Wilhelms-Universität Bonn, 53127 Bonn, Germany; 2 Research Group Clinical Neuroimaging, German Center for Neurodegenerative Diseases (DZNE), Bonn, Germany

**Keywords:** Cerebral aneurysm, Computer-assisted diagnosis, MRI, Diagnostic performance, Artificial intelligence

## Abstract

**Purpose:**

To evaluate the impact of an AI-based software trained to detect cerebral aneurysms on TOF-MRA on the diagnostic performance and reading times across readers with varying experience levels.

**Methods:**

One hundred eighty-six MRI studies were reviewed by six readers to detect cerebral aneurysms. Initially, readings were assisted by the CNN-based software mdbrain. After 6 weeks, a second reading was conducted without software assistance. The results were compared to the consensus reading of two neuroradiological specialists and sensitivity (lesion and patient level), specificity (patient level), and false positives per case were calculated for the group of all readers, for the subgroup of physicians, and for each individual reader. Also, reading times for each reader were measured.

**Results:**

The dataset contained 54 aneurysms. The readers had no experience (three medical students), 2 years experience (resident in neuroradiology), 6 years experience (radiologist), and 12 years (neuroradiologist). Significant improvements of overall specificity and the overall number of false positives per case were observed in the reading with AI support. For the physicians, we found significant improvements of sensitivity on lesion and patient level and false positives per case. Four readers experienced reduced reading times with the software, while two encountered increased times.

**Conclusion:**

In the reading with the AI-based software, we observed significant improvements in terms of specificity and false positives per case for the group of all readers and significant improvements of sensitivity and false positives per case for the physicians. Further studies are needed to investigate the effects of the AI-based software in a prospective setting.

**Supplementary Information:**

The online version contains supplementary material available at 10.1007/s00234-024-03351-w.

## Introduction

Cerebral aneurysms are estimated to be prevalent in 2% of the population and harbor the risk of severe morbidity and mortality in the event of rupture [1]. Both computed tomography (CT) and magnetic resonance imaging (MRI) are widely accepted as diagnostic tools in the detection of cerebral aneurysms [2]. mdbrain (Mediaire GmbH, Berlin, Germany) is a Conformité Européenne (CE)-marked and commercially available software designed to assist radiologists in reading MRI studies of the brain, like multiple sclerosis lesion detection [3], volumetry of the brain for quantification of cerebral atrophy, and of contrast-enhancing brain tumors, and aneurysm detection [4]. In a recent external validation study, the sensitivity of mdbrain in terms of aneurysm detection was reported to be 70% with a number of false positive results of 0.1 per case, with detection rates for saccular aneurysms of 76.6% (up to 100.0%, if the aneurysm diameter was 5 mm or larger) and low detection rates for fusiform (33.3%) or thrombosed (16.7%) aneurysms [4]. However, the software was not designed as an independent reader but as a tool to assist radiologists in their daily routines. Among others, artificial intelligence (AI)-based softwares have been shown to be of use in the detection of breast cancer [5] on digital mammograms or pulmonary nodules on CT scans [6] or chest radiographs [7]. Numerous AI-based softwares have been introduced and validated for the detection of cerebral aneurysms, both for CT imaging [8–10] and for MR imaging [11–17]. Also, recent studies have shown that AI-augmented reading of CT angiography (CTA) or time of flight MR angiography (TOF-MRA) may increase readers’ performances in terms of cerebral aneurysm detection [9, 18]. Our hypothesis was that the use of the software would improve the detection rates of raters with varying degrees of experience for cerebral aneurysms on TOF-MRA while decreasing the readers’ reading times.

## Materials and methods

Institutional review board approval was obtained for this retrospective study, and the need for written informed consent was waived.

No statistical power analysis was performed. To ensure that a sufficient number of aneurysms would be present in the dataset, a full-text research of our radiology information system (RIS) for known aneurysm cases was performed. The cases that were found in the full-text research were reviewed by a neuroradiologist and were then added to a set of consecutive MRI studies that were acquired at our institution and that included a TOF-MRA, resulting in a dataset of 186 MRI studies in total. The fact that most patients with cerebral aneurysms are referred to our institution with external imaging and that external images are rejected by our version of mdbrain, as well as the fact that most emergency imaging is done with CT at our institution, prevented us from including a larger number of aneurysms in our study.

The imaging technique and parameters were the same as previously described [4]. In brief, the imaging studies were acquired using two clinical 3 T scanners (Achieva, Philips Healthcare; Discovery, GE Healthcare) and one clinical 1.5 T scanner (Achieva, Philips Healthcare), using routine protocols of our institutions. For the acquisition of 3D TOF images, TR ranged from 19.33 to 20.12 ms and TE ranged from 3.68 to 3.80 ms. The slice thickness was 1 mm, and the increment was 0.5 mm. The field of view and matrix size were chosen according to the patient’s characteristics by the radiology technician. The imaging studies were reviewed by two neuroradiologists with 8 years of experience and 15 years of experience, respectively, in reading MR imaging studies of the brain, for the presence of cerebral aneurysms under full consideration of the patient’s medical records, previous and follow up imaging including digital subtraction angiography (DSA). In cases with differing results between the two readers, a consensus was reached to establish a reference standard.

Details of the training of the AI model have been published before and can be found in supplemental material S1. The imaging studies were analyzed by the artificial intelligence-based software mdbrain, version 4, and written reports as well as annotated series were automatically created by the software and imported to the institute’s Picture archiving and communication system (PACS). For each patient, two sets of hanging protocols were created in the institute clinical PACS viewer (Deep Unity, Dedalus Healthcare Group AG, Bonn, Germany), one of which included axial TOF images, maximum intensity projection (MIP) images of the TOF-MRA, and the findings of the software, and the second with only the above mentioned TOF images but without the findings of the software. Also, an Excel sheet (Excel, Microsoft Corporation, Redmond, WA, USA) was created which contained a schematic image of the circle of Willis with checkboxes at typical locations for the presence of cerebral aneurysms. The findings reported in the Excel sheet were automatically exported to an Excel table, and the reading time was measured by calculating the time when the reading was started and the time when it was finished.

Six readers (three medical students with no experience in image interpretation, one neuroradiology resident with 2 years of experience in diagnostic neuroradiology, one radiologist with 6 years of experience in diagnostic radiology and neuroradiology, and one neuroradiologist with 12 years of experience in diagnostic neuroradiology) were asked to review the imaging studies using the hanging protocols and to report their findings in the above mentioned Excel sheet. The readers were only allowed to review the images included in the respective hanging protocol, but they were allowed to create multiplanar reconstructions when needed. The readers had no knowledge of the original reports, the patient’s medical histories, or prior follow up imaging. First, the readers reviewed the hanging protocols including the software’s reports. After a washout period of at least 6 weeks, they read the imaging studies again but now without the assistance of the software.

**Fig. 1:**
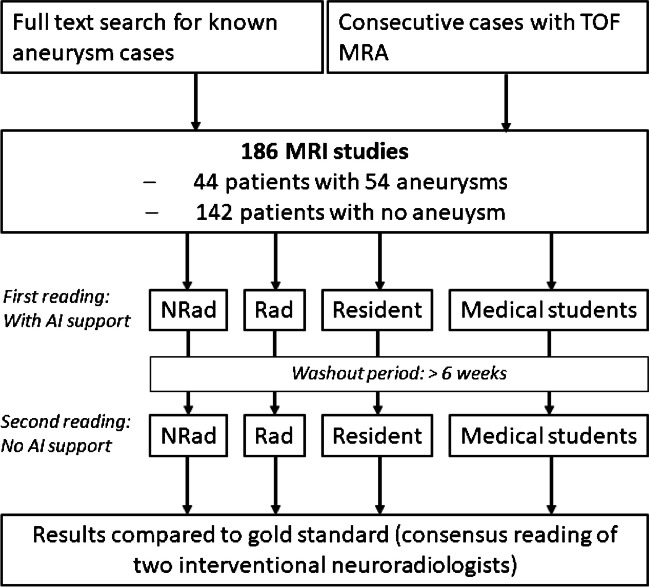
Flow chart of the study design. NRad neuroradiologist, Rad radiologist

Statistical analyses were performed with R statistical and computing software, Version 4.0.3 (http://www.r-project.org/) and R Studio, Version 1.2.5033 (http://rstudio.org/download/desktop). On the patient level, sensitivity and specificity were calculated for each reader with and without the use of the software. Only cases where all aneurysms were detected by the reader without any false positive findings were counted as true positives. Cases where both the reader and the reference standard reported no aneurysm were counted as true negatives. When at least one false positive finding was reported, the case was counted as false positive. When at least one aneurysm was missed by the reader, the case was counted as false negative. We also measured the sensitivity on lesion level, the rate of false positives per case, and the reading times. To avoid misinterpretation of aneurysms that were correctly detected by the readers, but with a false localization as false findings, we summarized aneurysms originating from the internal carotid artery (ICA), the carotid terminus, and the posterior communicating artery (PcoA) as ICA aneurysms; and aneurysms originating from the basilar artery, the basilar tip, the distal vertebral arteries, the posterior inferior cerebellar arteries (PICA), anterior inferior cerebellar arteries (AICA), superior cerebellar artery (SCA) or posterior cerebral artery (PCA) as posterior circulation aneurysms. The readers’ findings with and without AI support were compared with McNemar’s test, and the reading times of each reader with and without the use of the AI software were compared by using the Mann–Whitney *U* test. Normal distribution was evaluated by using the Shapiro–Wilk test. The level of statistical significance was set at *p* = 0.05. The readers’ diagnostic performances compared to the diagnostic reference standard were evaluated using confusion matrices. The study design is summarized in Fig. 1.

## Results

One hundred eighty-six patients were included in the study, 106 (57%) of the individuals were women, and 80 were men. The mean age was 58.4 years (median 62 years, range 18–95 years). One hundred thirty-six patients (73.1%) were scanned at 3 T, and the remaining 50 patients were scanned at 1.5 T. Five patients were scanned using a clinical GE scanner (Discovery, GE Healthcare), while the remaining 181 patients were scanned using two clinical Philips scanners (3 T and 1.5 T Achieva, Philips Healthcare). Fifty-four aneurysms were reported by the diagnostic reference standard. Fourty-four patients (23.7%) had at least one aneurysm, and ten patients (18.6%) had two aneurysms. The mean aneurysm’s largest diameter was 7.3 mm (median 4.1 mm, range 1.3–45.4 mm). Fifty-one aneurysms (94.4%) were saccular aneurysms, and the remaining three aneurysms were fusiform aneurysms. Six aneurysms (11.1%) showed signs of thrombosis. Twenty-four aneurysms (44.4%) were proved by DSA. Thirty-seven (68.5%) of the aneurysms were correctly detected by the software. The software reported 25 false positive findings (0.1 false positives per case). The patient and aneurysm characteristics are summarized in Table 1.

**Table 1:** Patient and aneurysm characteristics

Patient characteristics	No	%
Female	106	57.0
Male	80	43.0
Age		
Mean	58.4	
Median	62	
Range	18–95	
Positive for 1 aneurysm	44	23.7
Positive for > 1 aneurysm	10	18.6
Field strength		
3 T	136	73.1
1.5 T	50	26.9
Aneurysm characteristics		
Saccular	51	94.4
Fusiform	3	5.6
Signs of thrombosis	6	11.1
DSA proved	24	44.4
Detected by AI	37	68.5
Measurements		
Mean largest diameter	7.3	
Median largest diameter	4.1	
Range of diameters	1.3–45.4	
Localization		
ICA	28	51.9
ICA terminus	1	1.9
PCoA	7	13.0
C4–C6	20	37.0
ACA	8	14.8
ACoA	7	13.0
A1	1	1.9
MCA	11	20.4
Posterior circulation	7	13.0
Basilar tip	1	1.9
Basilar artery	2	3.7
SCA	2	3.7
PICA	1	1.9
V4	1	1.9
Total	54	100.0

*AI* artificial intelligence, *ICA* internal carotid artery, *PCoA* posterior communicating artery, *ACA* anterior cerebral artery, *ACoA* anterior communicating artery, *A1* A1 segment of the anterior cerebral artery, *MCA* middle cerebral artery, *SCA* superior cerebellar artery, *PICA* posterior inferior cerebellar artery, *V4* V4 segment of the vertebral artery

**Fig. 2:**
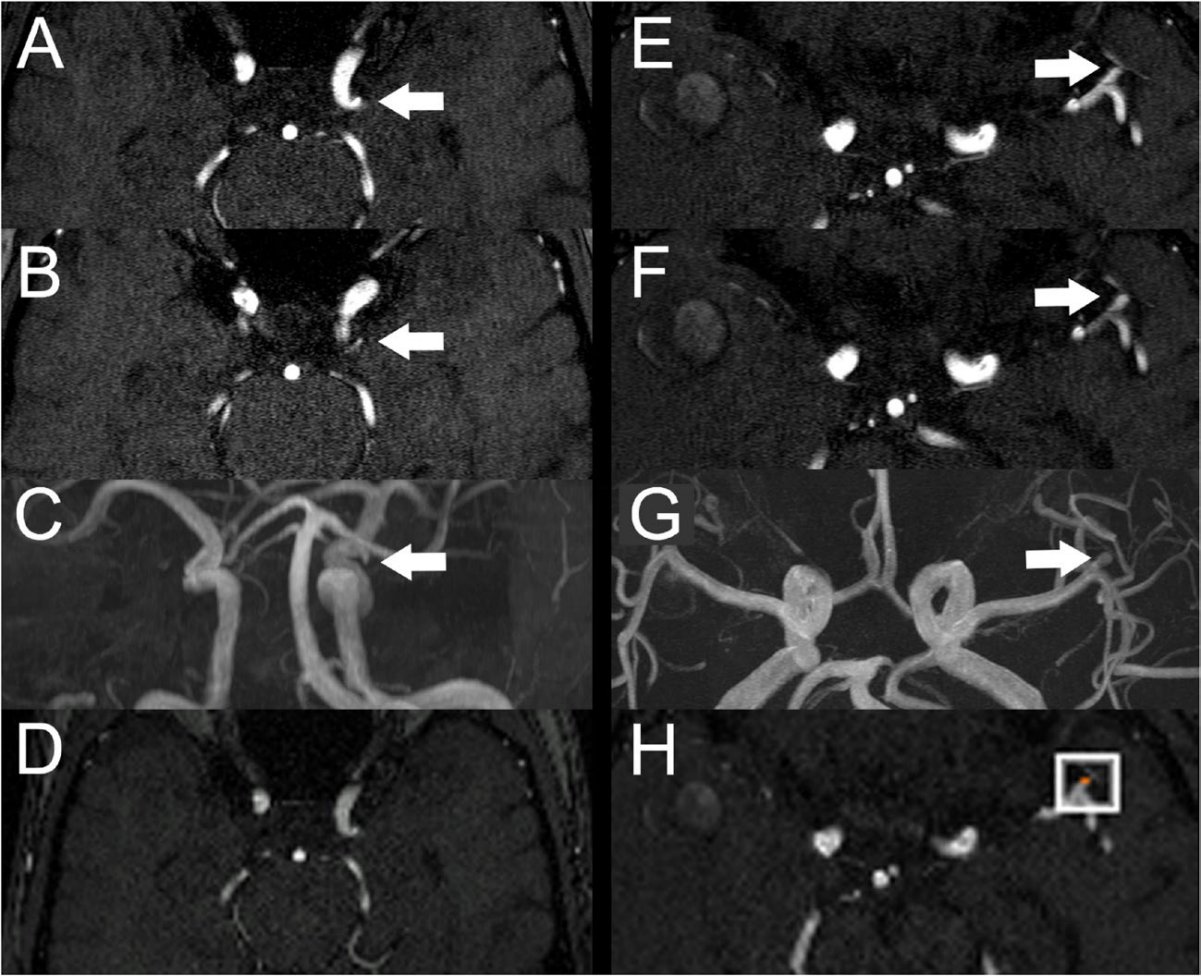
**A**–**D** Example of a false positive finding reported by the neuroradiologist. **A**, **B** Axial TOF image with infundibular origin of a small artery from the left ICA (arrows) reported as an aneurysm by the neuroradiologist in the reading without AI but reported as negative in the reading with AI. **C** MIP image of the same study with the infundibular origin highlighted (arrow). **D** Axial reconstruction of TOF-MRA created by the algorithm with no aneurysm detected. **E**–**G** Example of a left MCA aneurysm missed by the resident in the reading without AI but correctly reported in the reading with AI. **E**, **F** Axial TOF images with small left MCA aneurysm (arrows). **G** MIP image of the same study with the left MCA aneurysm highlighted (arrow). **H** Axial reconstruction of the TOF images created by the software with the aneurysm highlighted by a white box

**Fig. 3:**
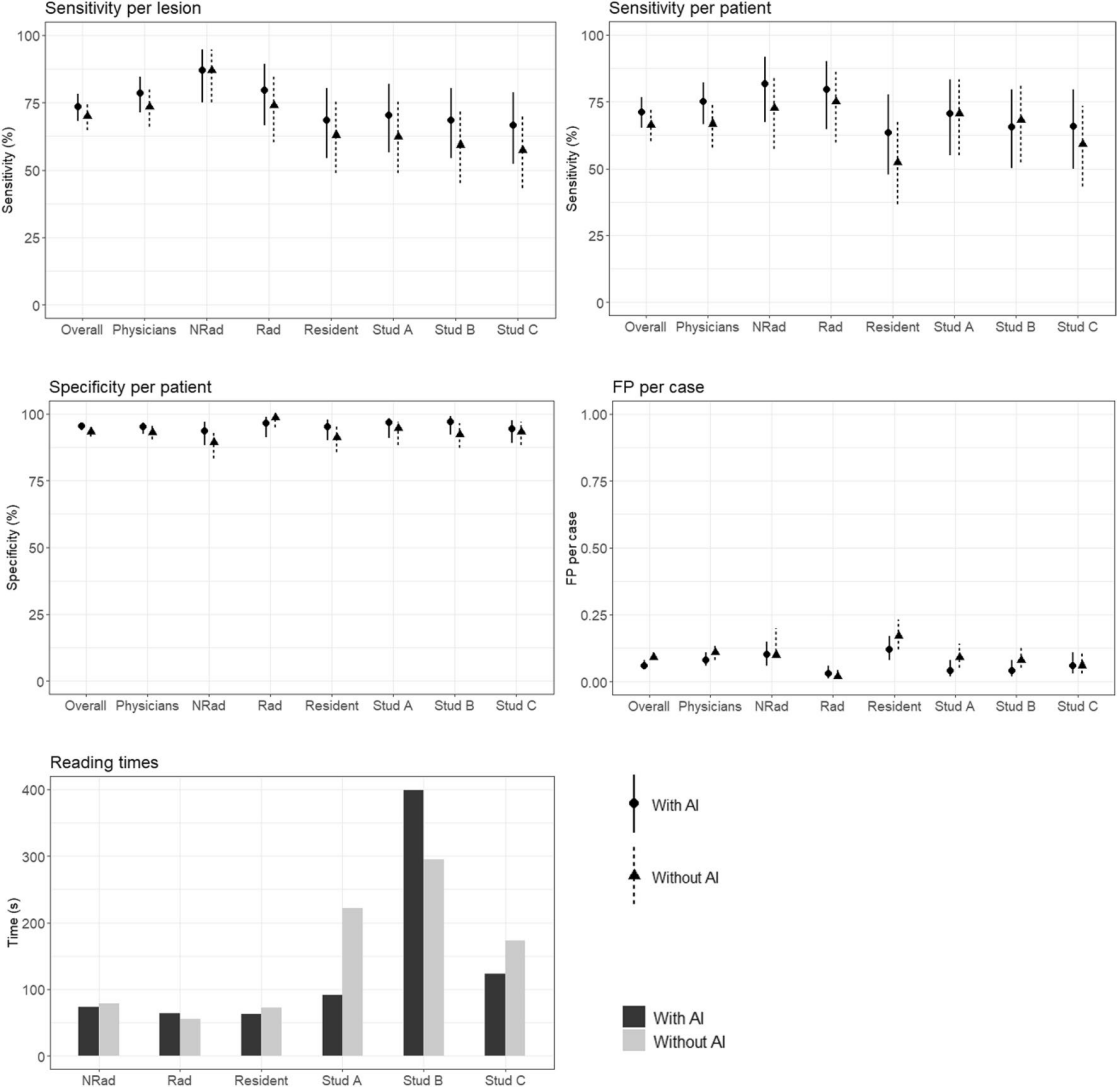
Diagnostic performance and reading times. NRad neuroradiologist, Rad radiologist, Stud student

We found significant differences in overall performance for specificity (95.4% with AI vs. 93.3% without AI, *p* = 0.04) and for false positives per case (0.06 with AI vs. 0.09, p = 0.005). For the subgroup of physicians, we found significant differences in the diagnostic performance for sensitivity on lesion level (78.4% with AI vs. 73.5% without, *p* = 0.03), sensitivity on patient level (75.0% with AI vs. 66.7% without AI, *p* = 0.04), and false positives per case (0.08 with AI vs. 0.11 without, *p* = 0.01). We found no statistically significant differences in the diagnostic performance of each individual reader. Figure 2 shows example images of false positives and false negatives alongside with the findings of the algorithm. The overall diagnostic performance, the physicians’ diagnostic performance as well as the diagnostic performances of each reader are summarized in Table 2 and visualized in Fig. 3.

**Table 2:** Diagnostic performance of the different readers with and without AI

Reader	Sensitivity lesion level (%)			Sensitivity patient level (%)			Specificity patient level (%)			False positives per case		
	With AI	Without AI	*p*	With AI	Without AI	*p*	With AI	Without AI	*p*	With AI	Without AI	*p*
Overall	73.5 (68.3–78.2)	70.1 (64.8–75.0)	0.27	71.2 (65.3–76.6)	66.3 (60.2–72.0)	0.12	95.4 (93.8–96.7)	93.3 (91.4–94.9)	0.04	0.06 (0.05–0.08)	0.09 (0.08–0.11)	0.005
Physicians	78.4 (71.3–84.5)	73.5 (66.0–80.1)	0.03	75.0 (66.7–82.1)	66.7 (57.9–74.6)	0.04	95.1 (92.6–96.9)	93.2 (90.4–95.4)	0.2	0.08 (0.06–0.11)	0.11 (0.08–0.14)	0.01
Neuroradiologist	87.0 (75.1–94.6)	87.0 (75.1–94.6)	0.37	81.8 (67.3–91.8)	72.7 (57.2–85.0)	0.39	93.7 (88.3–97.1)	89.4 (83.2–94.0)	0.18	0.1 (0.06–0.15)	0.1 (0.09–0.20)	0.06
Radiologist	79.6 (66.5–89.4)	74.1 (60.3–85.0)	0.61	79.5 (64.7–90.2)	75.0 (59.7–86.8)	0.62	96.5 (91.2–98.8)	98.6 (95.0–99.8)	0.37	0.03 (0.01–0.06)	0.02 (0.01–0.05)	1
Resident	68.5 (54.4–80.5)	63.0 (48.7–75.7)	0.13	63.4 (47.8–77.6)	52.3 (36.7–67.5)	0.13	95.1 (90.1–97.8)	91.2 (85.7–95.6)	0.23	0.12 (0.08–0.17)	0.17 (0.12–0.23)	0.05
Student A	70.4 (56.4–82.0)	62.3 (48.7–75.7)	0.39	70.5 (54.8–83.2)	70.5 (54.8–83.2)	1	96.8 (91.0–98.4)	94.6 (88.3–97.1)	0.58	0.04 (0.02–0.08)	0.09 (0.05–0.14)	0.45
Student B	68.5 (54.4–80.5)	59.3 (45.0–72.4)	1	65.6 (50.1–79.5)	68.2 (52.4–81.4)	1	97.2 (92.3–99.2)	92.3 (87.4–96.6)	0.08	0.04 (0.02–0.08)	0.08 (0.05–0.13)	0.18
Student C	66.7 (52.3–78.9)	57.4 (43.2–70.1)	0.36	65.9 (50.0–79.5)	59.1 (43.2–73.4)	0.61	94.4 (89.2–97.5)	93.4 (88.3–97.1)	1	0.06 (0.03–0.11)	0.06 (0.03–0.11)	1

Data in parentheses are 95% confidence intervals. *AI* artificial intelligence

The mean reading time was 136 s (range 19–872 s) with AI and 149 s (range 29 s–500 s) (*p* < 0.001). For the neuroradiologist, the mean reading time was 73 s (range 30–158 s) with AI and 79 s (range 45–145 s) without (*p* = 0.003). For the radiologist, the mean reading time was 64 s (range 25–253 s) with AI and 55 s (range 29–91 s) without (*p* < 0.001). For the resident, the mean reading time was 63 s (range 19–186 s) with AI and 72.1 s (range 31–198 s) without (*p* < 0.001). For student A, the mean reading time was 398.8 s (range 201–872 s) with AI and 295 s (range 177–487 s) without the support of the software (*p* < 0.001). For student B, the mean reading time was 92 s (range 41–659 s) with AI and 222 s (range 123–463 s) without (*p* < 0.001). For student C, the mean reading time was 123 s (range 27–704 s) with AI and 173 s (86–500 s) without (*p* < 0.001). Reading times are visualized in Fig. 3.

## Discussion

In this single-center reading study, we compared the diagnostic performance of readers with different levels of experience for the detection of cerebral aneurysms on TOF-MRA with and without the aid of the commercially available AI software mdbrain. No significant effects on the diagnostic performances could be found for the individual readers, but for the group of physicians, we found significant differences between the readings for sensitivity both on the lesion and on the patient level and for false positive findings per case, each in favor of the use of the software. Also, we found a moderate but statistically significant reduction in reading times for the majority of readers, while the radiologist and one of the students showed a significant increase in mean reading times. The latter, we attribute to a training effect of the unexperienced reader between the first and second reading.

There are only few studies that examined the influence of AI software on the detection rates of cerebral aneurysms. Sohn et al. performed a similar study on their algorithm to determine its influence on the diagnostic performance of a neuroradiologist, a radiology resident, a neurologist, and a neurosurgeon [18]. They found significant overall improvement for sensitivity on the patient level and sensitivity on the lesion level, comparable to the results that we found for the subgroup of physicians. Different from our findings, the investigators found a significant increase in the number of false positive findings per case with the use of their software. We attribute this positive effect of mdbrain to its intrinsically low rate of 0.1 false positive findings per case, although we lack an explanation why the software of Sohn et al. had no similar effect despite a comparable number of 0.12 false positives per case. While the investigators were able to show significant improvements for the individual readers in terms of sensitivity, we were not. This difference may be due to the lower number of cases in our dataset (186 MR studies with 54 positive findings vs. 332 MR studies with 169 aneurysms), but also due to the fact that Sohn et al. performed the reading with AI assistance after the reading without assistance with the risk of a training effect, especially for the inexperienced readers, a bias that we tried to avoid by performing the reading without AI after the reading with AI assistance. Similar to us, Sohn et al. found positive effects on reading times for the individual readers. Contrary, Müller et al. reported an increase of reading times for a radiology resident using an AI software designed to aid in the reading of chest CTs [19] which was, however, not statistically significant. Therefore, although it is rarely doubted that AI assistance will have positive effects on radiologists’ diagnostic performance and workload, it is crucial to critically evaluate each software for its use for the individual readers and its integration in the working environment, as standalone softwares without PACS integration are at risk to slow down the process of image interpretation [20]. Mdbrain’s reports are sent to the PACS and are available as DICOM images that can be viewed like any MR sequence, which we regard as a pragmatic approach to PACS integration.

It is well known that increasing the sensitivity of a diagnostic test will usually result in an increase of false positive findings. Contrary, in our study, we observed both an increase of sensitivity for the subgroup of physicians and a decreased rate of false positive findings when using the software. We hypothesize that the low rate of false positive findings reported by the algorithm may have reassured readers to dismiss suspected findings while adding a certain number of true positive findings to the readings. While a further reduction of false positives per case does not seem necessary for mdbrain, we believe that improving the sensitivity of the software alone would maybe result in stronger effects on the diagnostic performance of readers. Kuwabara et al. reported a decrease of false positives by further tuning their algorithm while maintaining an acceptable sensitivity of 90.0% in an epidemiological setting [21]. Similar attempts may be made for mdbrain to increase sensitivity while maintaining the low rates of false positive findings, depending on the setting it will be used for. While the main task for an AI algorithm in an epidemiological setting will be a low rate of false negatives (at an acceptable rate of false positive findings to reduce the workload of supervising physicians), a lower sensitivity may be acceptable in an everyday clinical setting.

## Limitations

We acknowledge that our study has several limitations. First, its retrospective nature may limit its interpretability for the daily clinical work, and our cohort does not reflect an everyday patient cohort. Second, no statistical power analysis was performed to determine a sufficient sample size, limiting the statistical significance for the individual readers. Third, we cannot exclude training effects in the second reading, although we tried to minimize these effects by leaving a washout period and by changing the order of patients between the first and second reading. Fourth, the reading situation was artificial and the only task was to detect aneurysms, which do not fully reflect the daily clinical routine. Fifth, different from Sohn et al. [18], we did not include clinicians other than (neuro-) radiologists in our study, limiting our results to this group of readers. We deliberately excluded specialties other than radiologists, as in the German healthcare system, MRI studies are mainly read by radiologists, and if reported to be negative, they will most likely not undergo second readings, especially in the setting of incidental findings. Sixth, images used for algorithm training and internal validation were all obtained using Philips scanners, like the majority of the images used for our study. Thus, our study would be more generalizable if our dataset were more diverse in terms of the range of device manufacturers represented. Seventh, we did not evaluate the effects of the software on the daily workflow of radiologists apart from reading times. The actual integration of the software will need to be investigated in more prospective settings. Eighth, although we found both an improvement in sensitivities and a reduction of false positive findings, we did not further investigate the potential effects of the software on patient management and patient outcomes. The increase of sensitivity alongside with a decrease of false positives may result in both a reduction of missed clinically significant aneurysms and a reduction of unnecessary further examinations, like follow up scans or DSA, potentially reducing complications and costs to the healthcare system. However, the question of patient outcomes and management needs to be addressed in further studies.

## Conclusion

In conclusion, we were able to demonstrate positive effects on the diagnostic performance of a group of readers with different amounts of experience in the detection of cerebral aneurysms, while we were not able to show significant effects for the individual readers. Also, we were able to show that the use of the software reduced the reading times for the majority of readers. Further studies with a larger number of cases are needed to investigate the effects of the use of mdbrain on the performance of individual readers and its effects on the diagnostic performance of readers in a prospective setting.

### Supplementary Information

Below is the link to the electronic supplementary material.

Supplementary file1 (DOCX 15 kb)

## Data Availability

Data generated or analyzed during the study are available from the corresponding author by request.

## Abbreviations

AI: Artificial intelligence
CNN: Convolutional neural network
ICA: Internal carotid artery
MCA: Middle cerebral artery
PCoA: Posterior communicating artery
SCA: Superior cerebellar artery
PCA: Posterior cerebral artery
