# Supplementary material for: Impact of an AI software on the diagnostic performance and reading time for the detection of cerebral aneurysms on time of flight MR-angiography
Source: Neuroradiology. 2024 Apr 15;66(7):1153–60. doi: 10.1007/s00234-024-03351-w (PMC11150207; doi:10.1007/s00234-024-03351-w)
Supplement: Supplementary file 1 — Supplementary file1 (DOCX 15 kb) [file 234_2024_3351_MOESM1_ESM.docx]

**Supplemental materials**

S1 Details on the training of the AI algorithm

As described before, the segmentation algorithm of mdbrain uses a 3D convolutional neural network with a U-NET architecture. The training data set included a total of 93 saccular aneurysms and no fusiform aneurysms. The aneurysms in the training data set were localized at the anterior communicating artery (17%), the A2 segment of the anterior cerebral artery (10%), the C6 segment of the internal carotid artery (20%), the C7 segment of the internal carotid artery (22%), the middle cerebral artery (20%), and the basilar artery (9%) [4].
